# Defect dependent electronic properties of two-dimensional transition metal dichalcogenides (2H, 1T, and 1T′ phases)†

**DOI:** 10.1039/d4cp04017a

**Published:** 2024-12-06

**Authors:** Berna Akgenc Hanedar, Mehmet Cengiz Onbaşlı

**Affiliations:** a Department of Physics, Kirklareli University Kirklareli 39100 Turkey berna.akgenc@klu.edu.tr +90 288 246 17 33 +90 288 246 17 34; b Department of Physics, Koc University, Rumelifeneri Yolu Sariyer 34450 Istanbul Turkey bhanedar@ku.edu.tr; c Department of Electrical & Electronics Engineering, Koc University, Rumelifeneri Yolu Sariyer 34450 Istanbul Turkey monbasli@ku.edu.tr +90 212 338 1711

## Abstract

Transition metal dichalcogenides (TMDs) exhibit a wide range of electronic properties due to their structural diversity. Understanding their defect-dependent properties might enable the design of efficient, bright, and long-lifetime quantum emitters. Here, we use density functional theory (DFT) calculations to investigate the 2H, 1T, and 1T′ phases of MoS_2_, WS_2_, MoSe_2_, WSe_2_ and the effect of defect densities on the electronic band structures, focusing on the influence of chalcogen vacancies. The 2H phase, which is thermodynamically stable, is a direct band gap semiconductor, while the 1T phase, despite its higher formation energy, exhibits metallic behavior. 1T phases with spin–orbit coupling show significant band inversions of 0.61, 0.77, 0.24 and 0.78 eV for MoS_2_, MoSe_2_, WS_2_ and WSe_2_, respectively. We discovered that for all four MX_2_ systems, the energy difference between 2H, 1T and 1T phases decreases with increasing concentration of vacancies (from 3.13% to 21.88%). Our findings show that the 2H phase also has minimum energy values depending on vacancies. TMDs containing W were found to have a wider bandgap compared to those containing Mo. The band gap of 2H WS_2_ decreased from 1.81 eV (1.54 eV with SOC included) under GGA calculations to a range of 1.37 eV to 0.79 eV, while the band gap of 2H MoSe_2_ reduced from 1.43 eV (1.31 eV with SOC) under GGA to a range of 0.98 eV to 0.06 eV, depending on the concentration. Our findings provide guidelines for experimental screening of 2D TMD defects, paving the way for the development of next-generation spintronic, electronic, and optoelectronic devices.

## Abstract 2

Transition metal dichalcogenides (TMDs) exhibit a wide range of electronic properties due to their structural diversity.

## Graphical abstract

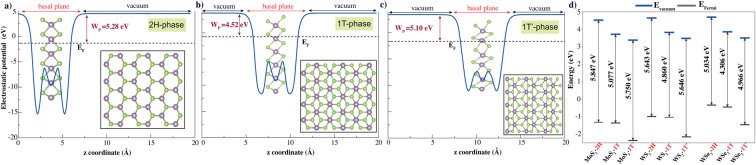

2D transition metal dichalcogenides (TMDs)^1–3^ provide rich materials for physics and materials science that are not available in bulk or other quantum confined materials. These platforms may help in discovering fundamental new spin physics and quantum information processing.^4,5^ The ability to control the band gap,^6^ electrostatically or chemically tune the Fermi level,^7^ and induce defects that could detect or emit (polarized) single photons^8^ enables researchers to investigate a wide range of interactions such as single spin-phonon,^9–11^ spin-magnetic field,^12^ spin-electric field, defects with different external baths such as superconductor,^13^ ferromagnetic,^14^ or piezoelectric substrates.^15^ These interactions are strongly correlated^16^ with the intrinsic defects (vacancies, anti-site defects, substitutional dopants, grain boundaries and finite size edge effects) and extrinsic disorders (strain, substrate termination, terraces, and vicinal surfaces). The point defects may include vacancy centres (chalcogen or transition metal sites in TMDs), substitutional defects, interstitial defects, and anti-site defects.^17^

These defects may cause phonon–magnon, phonon–exciton and magnon–magnon scattering due to the broken translational symmetry and the defect-driven semi-metallic or magnetic band structures. Understanding these interactions may help discover a range of ultrafast transport and photon emission mechanisms that may assist in engineering two-level systems with extended decoherence time and bright emission.^18^ Their applications include quantum sensing, enhanced super-resolution microscopy,^19^ and quantum communication.^20^

Point defects form naturally during growth but their effects on electronic band structures, and optical and magnetic properties are not well understood especially for the 2H, 1T and 1T′ phases.^21,22^ These different phases of 2D TMD layers and their defect characteristics strongly affect their electronic and optical properties.^23–26^ Bottom-up growth techniques such as molecular beam epitaxy (MBE) processes need to be optimized to obtain pure and desired phases of TMD layers with controlled defect density.^27,28^ Electronic band structural and optical characteristic signatures of the phases and defect types of 2D TMD layers must be understood for obtaining the optimal MBE recipes. Thus, a computational technique must be used to elucidate these phase and defect type-dependent signatures of TMD layers.

TMDs, whose formula unit is MX_2_, where M represents a transition metal and X is a chalcogen atom, consist of a transition metal layer sandwiched between two chalcogen layers as a unit layer. The intralayer interactions are based on covalent bonds, while the interlayer interaction between two slabs takes place *via* van der Waals interactions that stabilize the bulk material. By considering the arrangement of atoms along the *c*-axis in the monolayer of the sandwich structure, the stacking sequence AbA is for the 2H phase and AbC is for the 1T phase, where A and C denote chalcogen atoms and b denotes the metal atom. In the 1T structure, the distorted transition metal atoms formed a period doubling 2 × 1 structure consisting of 1D zigzag chains. TMDs exhibit different phases characterized by varying coordination structures (trigonal, octahedral and distorted octahedral) and stacking orders, resulting in 2H, 1T and 1T′-phases;^29–31^ these possess unique electronic properties. For instance, MoS_2_, MoSe_2_, WS_2_ and WSe_2_ systems have been shown to crystallize in the 2H phase under ambient conditions. 2H phase TMDs have been extensively studied for applications including catalysts,^32,33^ super-capacitors,^34^ and batteries,^35^ and for other energy-related applications^36^ due to the outstanding catalytic, optical and electronic performance. Although 1T phase of TMDs has higher formation energy compared with 1T′ and 2H phases, forming a metastable (semi)metallic 1T phase enhances charge transfer efficiency in many energy-related and electrochemical processes.^37–40^ The metastable nature of the 1T phase allows it to spontaneously convert into the 2H phase under the stimulation of external conditions.^41,42^ Despite these interesting properties, a comprehensive understanding of the 2H, 1T and 1T′ phases of pristine TMDs is lacking. In addition, since these phases commonly form with defects in experiments, understanding the effect of chalcogen vacancies on the structural and electronic band structure properties of TMDs is needed for applications.

Here, we use DFT to calculate the structural, electronic and phonon mode properties of pristine 2H, 1T and 1T′ phases of MoS_2_, WS_2_, MoSe_2_, and WSe_2_ TMD single layers. Next, we also investigate and present the effect of increasing chalcogen vacancy concentrations in each TMD phase on their structural and electronic properties. In Section 1, we describe and justify the details of our computational methodology. In Section 2.1, we first present the equilibrium structural electronic band properties of the defect-free and pristine 2H, 1T and 1T′ phases of XY_2_ (transition metal X = Mo, W; chalcogen atom Y = S, Se). In Section 2.2, we present the defect density and phase type dependence of the structural, electronic, and magnetic properties of the TMD monolayers. In the Conclusion section, we highlight the emergence of a strong dependence of the electronic band structure on the defect density and orientations. Finally, we suggest guidelines for experimental screening of 2D defects and their structural properties based on the outcomes of our DFT results.

## 1. Computational methodology

The spin-polarized first-principles calculations were performed within the framework of DFT implemented in the Vienna *ab initio* simulation package (VASP).^43,44^ A plane-wave basis set with the kinetic energy cut-off *ħ*^2^(*k* + *G*)^2^/2*m* = 500 eV was used.^45^ The exchange–correlation term was described with generalized-gradient approximation (GGA-PBE).^46^ The van der Waals (vdW) interaction was included by using the DFT-D2 method of Grimme.^47^ All structures were treated using periodic boundary conditions. The Brillouin zone (BZ) integration was performed in *k*-space within the Monkhorst–Pack scheme using the (16 × 16 × 1) and (4 × 4 × 1) special mesh points for the (1 × 1) and (4 × 4) pristine and chalcogen vacancy cells with different concentrations, respectively. To prepare the chalcogen vacancy concentration models, pristine single monolayers with a supercell of lateral size (4 × 4) were constructed (16 X = Mo/W atoms and 32 Y = S/Se atoms for pristine XY_2_ crystal), and some of the Y = S/Se atoms of the model were increasingly removed in various configurations. The percentage of S/Se-vacancy can be defined as the number of vacancies divided by the total S/Se atoms. For instance, three S-vacancies (3V) correspond to a sulfur vacancy percentage of 9.38%, associated with a specific density. Multiple different configurations were considered and classified as “cluster”, “diagonal”, and “random”; based on the configuration of the S/Se-vacancies. Previous DFT calculations showed that “clustering” type of growth is thermodynamically favored.^48,49^ The significant rearrangements were observed for the range of S/Se-vacancies considered in the current study when fully relaxing the unit cell and atomic positions. We have considered the minimum energy configurations. The lattice constants and atoms were optimized without any constraint until the energy difference between two sequential steps was less than 10^−5^ eV, and the maximum force on atoms was smaller than 10^−3^ eV Å^−1^. The maximum pressure on the unit cell was less than 1 kbar. A vacuum space of ∼15 Å was inserted along the *z*-direction to avoid the fictitious interactions generated due to periodic boundary conditions. The phononic properties were calculated in terms of the off-resonant Raman activities of the phonon modes at the Γ point. For this purpose, the zone-centered vibrational phonon modes were calculated using the finite-difference method as implemented in VASP. Each atom in the primitive unit cell was initially distorted by 0.01 Å and the corresponding dynamical matrix was constructed. Then, the vibrational modes were determined by direct diagonalization of the dynamical matrix. The kinetic energy cutoff for plane-wave expansion was increased to 800 eV and the global break condition for the electronic SC-loop was specified smaller than 10^−8^ eV with a *k*-point set of 24 × 24 × 1 in the case of Raman calculations. Once the accurate phonon mode frequencies were obtained at the Γ point, the change of the macroscopic dielectric tensor was calculated with respect to each vibrational mode to obtain the corresponding Raman activities. *Ab initio* molecular dynamics (AIMD) simulations were carried out to examine the thermal stability of the 2H phase MoS_2_ monolayers as a function of the concentration of chalcogen vacancy at 300 K with a total simulation time of 3 ps.

## 2. Results and discussion

### 2.1. Pristine monolayer 2H, 1T and 1T′ TMD phases

**Fig. 1:**
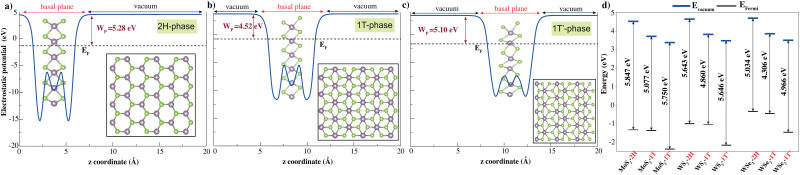
Plane-averaged electrostatic potential energy of pristine (a) 2H, (b) 1T and (c) 1T′ transition metal dichalcogenides along the normal direction. The Fermi level (*E*_F_) and work function (*W*_P_) are indicated by the black dashed line and claret red double-headed arrow, respectively. The top and side views of TMDs are also shown (Mo, W) and chalcogens (S, Se) are represented with purple and green spheres, respectively. (d) The work functions of all TMDs; the vacuum level *E*_vacuum_ and the Fermi level *E*_Fermi_ are depicted by solid lines in blue and black, respectively.

TMD layers studied here consist of a transition metal layer sandwiched between two chalcogen layers as a unit layer. The 2H phase is described with trigonal symmetry with *D*_3d_ (space group 194 or *P*6_3_/*mmc*). The 1T phase has octahedral coordination of the metal atom with *D*_3d_ (space group 164 or *P*3̄*m*1), while the octahedral coordination of the metal atom in the 1T′ phase is distorted with the space group *P*21/*m*. Fig. 1 shows the top and side views of the 2H, 1T and 1T′ monolayer phases. 2H monolayers exhibit mirror and inversion symmetry. 1T monolayer breaks the mirror symmetry of the 2H phase, while the 1T′ phase breaks both mirror and inversion symmetries of 2H monolayer. The symmetry breakings promote electronic band structure modifications. The work function is a crucial parameter for any electronic material, as it determines how the material interacts with external interfaces and governs charge transfer and transport across those interfaces. Studying the electrostatic potentials of TMD is also essential. Fig. 1d displays the electrostatic potential of pure monolayer XY_2_ (X = Mo, W; Y = S, Se). The work function is defined as the difference in energy level at vacuum and Fermi energy (WF = *E*_vacuum_ − *E*_Fermi_).

We first start with a discussion on the structural and electronic properties of pristine single layer 2H, 1T and 1T phases of XY2 (X = Mo, W; Y = S, Se) and then investigate their structural and electronic band structure differences from the defect cases. To prove the stability of pristine single layers of 2H, 1T and 1T phases of XY2 (X = Mo, W; Y = S, Se), we calculated their cohesive energies per pair of atoms, defined as follows:

1 *E*_coh_ = *pE*_Mo,W_ + *qE*_S,Se_ − *E*_XY_2_(X=Mo,W;Y=S,Se)_/(*p* + *q*)

where *E*_Mo,W_, *E*_S,Se_ and *E*_XY_2_(X=Mo,W;Y=S,Se)_ are the total energies of an isolated single transition metal atom: Mo and W are isolated single chalcogen atom: S and Se, *p* and *q* represent the number of particular atoms in pristine monolayer 2H, 1T and 1T′ phases of MoS_2_, MoSe_2_, WS_2_, and WSe_2_, respectively.

2 *E*_for_ = *E*_XY_2_(X=Mo,W;Y=S,Se)_ − *pμ*_Mo,W_ − *qμ*_S,Se_/(*p* + *q*)

here, *μ*_Mo_, *μ*_W_, *μ*_S_ and *μ*_Se_ represent the chemical potentials of Mo, W, S and Se atoms. They are obtained from their 3D bulk structures. The *μ*_atom_ is estimated from the equation given as *μ*_atom_ = *E*_bulk_/*N*, where *E*_bulk_ represents the total energy of bulk Mo, W, S, and Se atoms. N denotes the number of Mo, W, S, and Se atoms in their bulk form with space groups *Im*3*m̄*, *Im*3̄*m*, *Fddd*1 and *P*12_1_/*c*1 respectively.

After full optimization, the cohesive and formation energies, lattice constants and bond lengths of single layer 2H, 1T and 1T′ phase XY_2_ (X = Mo, W; Y = S, Se) are given in Table 1. The cohesive energies of pristine 2H, 1T and 1T′ phases of MoS_2_ were calculated as 5.63, 5.36 and 5.43 eV, respectively. Although the cohesive energies of the 2H, 1T and 1T′ polytypes are close to each other, the 2H phase is the most thermodynamically stable structure. After 2H, 1T′ has been found to be the second most stable phase. The 1T phase has the lowest cohesive energy and might be metastable.

**Table 1:** The calculated *E*_coh_ (eV per atom) is the cohesive energy; *E*_for_ (eV per atom) the formation energy; *a* (Å) and *b* (Å) are the optimized lattice constants; *d*_M–Y_ is the bond distances between the transition metal atom (Mo, W) and chalcogen atom (S, Se) for pristine single layer 2H, 1T and 1T′ phase XY_2_ (X = Mo, W; Y = S, Se) structures, respectively

Systems	*E*_coh_	*E*_for_	*a*, *b*	*d*_M–C_u_p_, *d*_M–C down_	*W*_P_
2H-MoS_2_	5.63	−0.79	3.19, —	2.41, —	5.84
1T-MoS_2_	5.36	−0.52	3.20, —	2.42, —	5.08
1T′-MoS_2_	5.43	−0.59	3.18, 5.67	2.47, 2.49	5.76
2H-MoSe_2_	5.15	−0.60	3.32, —	2.54, —	5.28
1T-MoSe_2_	4.92	−0.37	3.29, —	2.55, —	4.52
1T′-MoSe_2_	5.02	−0.46	3.29, 5.93	2.61, 2.63	5.10
2H-WS_2_	6.07	−0.52	3.18, —	2.41, —	5.64
1T-WS_2_	5.78	−0.23	3.22, —	2.43, —	4.86
1T′-WS_2_	5.90	−0.35	3.18, 5.65	2.39, 2.49	5.65
2H-WSe_2_	5.55	−0.29	3.33, —	2.54, —	5.03
1T-WSe_2_	5.30	−0.03	3.31, —	2.55, —	4.31
1T′-WSe_2_	5.44	−0.18	3.30, 5.85	2.60, 2.62	4.97

The structural difference between the 2H and 1T′ phases lies in their different symmetry configurations. The calculated energy differences (per formula XY_2_ unit) between pristine 2H and 1T/1T′ phases of TMDs. The energy needed for the phase transformation of 2H to 1T is more than 2H phase to 1T′ phase in all structures. For the 2H to 1T′ phase transformation, the energy differences are found to be 0.31 eV for WSe_2_, 0.41 eV for MoSe_2_, 0.49 eV for WS_2_ and 0.59 eV for MoS_2_. For the 2H to 1T phase transformation, the energy differences are found to be 0.69 eV for MoSe_2_, 0.76 eV for WSe_2_, 0.83 eV for MoS_2_ and 0.87 eV for WS_2_. Similar studies of the transition from the 2H phase to 1T′ phase of MoS_2_ were reported experimentally previously^50^ and under strain.^51^ To the best of our knowledge, the energy differences of 2H-to-1T transitions are presented here for the first time in the literature. Our results indicate that transforming the 2H phase of MoS_2_ into the 1T′ phase is energetically less favorable (requires 0.59 eV), while the 2H-to-1T′ transition in WSe_2_ requires less energy (0.31 eV) (Fig. S1, ESI†).

**Fig. 2:**
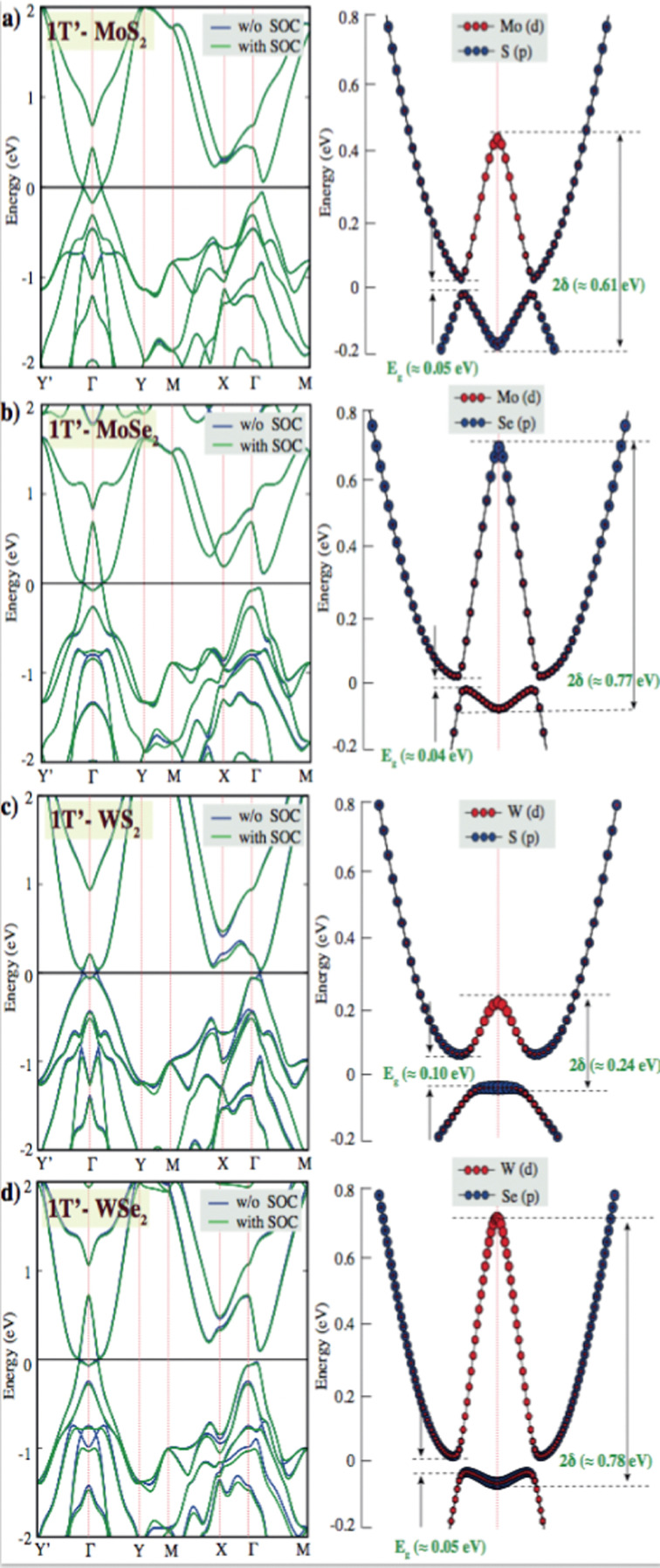
Band structure of pristine single layer 1T′ phase (a) MoS_2_, (b) MoSe_2_, (c) WS_2_, and (d) WSe_2_ PBE band structure is shown with a blue line and the PBE + SOC band structure is presented with a green line. The black line indicates the Fermi level at zero eV. *E*_g_ and 2*δ* are indicated fundamental and inverted gaps for PBE + SOC calculations.

Tunable electronic properties of materials are highly desirable from the perspective of potential device applications. We further investigated the electronic properties of the pristine single layer 2H, 1T and 1T′ phase XY_2_ (X = Mo, W; Y = S, Se) structures. The calculated band structures of 2H, 1T and 1T′ XY_2_ (X = Mo, W; Y = S, Se) shown in Fig. 2 are in good agreement with the previous reports. For group-VIB layered dichalcogenides, semiconducting properties are shown in their thermodynamically stable 2H phase.

The band structures of pristine single layers of the 2H phase for WSe_2_, MoSe_2_, WS_2_, and MoS_2_ have been calculated on the basis of PBE and PBE + SOC methods. The results indicate that the monolayer 2H phase of WSe_2_, MoSe_2_, WS_2_, and MoS_2_ behaves as direct band gap semiconductors at the K point. W sulfides and selenides have larger band gaps than their Mo counterparts. This is because a large part of the band gap arises from the crystal field splitting of the metal d states, and this is larger in W.^52^ The pristine single layer MoS_2_ has theoretical band gap values at the PBE and PBE + SOC levels that are found to be 1.61 eV and 1.56 eV, with the spin–orbit coupling effect reducing the band gap level by 0.05 eV. This phenomenon is also observed in other 2H-phase group-VIB layered dichalcogenides. 2H phases of TMDs are spin splitting of the electronic bands caused by spin–orbit coupling, which results from the absence of the inversion symmetry. Because SOC streams from electrostatic interactions and relativistic effects, the coupling effect for heavy elements is stronger, and thus the splitting of 0.27 eV for 2H-WS_2_ is larger than that of 0.05 eV in MoS_2_. The same splitting is observed, and that of 0.30 eV for 2H-WSe_2_ is larger than that of 0.12 eV in MoSe_2_. The intrinsic property of 2H-TMDs has been proven to provide a platform for exploring spin–valley polarization^53,54^ and designing spintronic devices. Valley degree of freedom is the *k*-space momentum observable that refers to the local minima in the energy band diagrams of semiconducting 2D materials.^55,56^ K-point minima appearing near the same energy levels allow for independent control of this degree of freedom for quantum information processing and storage especially in graphene, 2D TMD and other related materials. The realization of valleytronic material applications related to achieving broken inversion symmetry in the honeycomb lattice. TMDs have a structure resembling a honeycomb lattice, with their monolayer naturally exhibiting broken inversion symmetry and a direct band gap, making them ideal materials for valleytronic applications.^57,58^ In the band structures of the 1T′ phases of the TMD layers investigated, the valley degree of freedom emerged as another energy minimum between X and Γ points. These points might serve as a separate handle for valleytronic control of both spin and valley polarization indices. When these layers are introduced with defects with different concentrations, valleytronic control becomes much harder or nearly impossible as shown in the ESI,† Fig. S4, which indicates that the defect states alter the feasibility of manipulation of valley degree of freedom.

Besides stable 2H phases of TMDs, the metastable 1T and 1T′ phases also cause significant attention in the research area due to the newly proposed intriguing physical properties and promising applications in energy conversion and storage. For instance, while bulk 1T MoS_2_ crystals are demonstrated to undergo a superconducting transition at 4 K,^59^ 1T′ MoS_2_ and WTe_2_ monolayers have exhibited the quantum spin Hall effect.^60^ Moreover, 1T/1T′ MoS_2_ showed very high conductivity which makes them excellent electrocatalysts for hydrogen evolution and rechargeable batteries.^37^ According to the crystal field theory, the d orbitals of metals with trigonal coordination are classified into three groups, d_(*z*^2^)_, d_(*x*^2^−*y*^2^,*xy*)_ and d_(*xz*,*yz*)_, while the d orbitals with octahedral coordination form the degenerate d_(*z*^2^,*x*^2^−*y*^2^)_ and d_(*yz*,*xz*,*xy*)_ orbitals. 2H-phases TMDs showed that the d_*z*^2^_ orbital is completely filled and d_*xy*_ and d_*x*^2^−*y*^2^_ orbitals are empty. Configurations of W-6s^2^6p^4^ and Mo-5s^2^4d^4^ were treated as valence electrons, making the 2H phases of TMDs behave as a semiconductor. Due to 1T phases of TMDs are split into the configuration of e_g_ orbitals d_(*x*^2^−*y*^2^)_ d_(*z*^2^)_ over t_2g_ orbitals d_(*xy*)_, two electrons are filled in the t_2g_ state, the electronic structure of the T phases of TMDs leads to metallic phases and also various distortions that can lower the degeneracy and in some cases result in new semiconducting and semimetallic phases.

Reports have shown theoretically and experimentally that the metallic 1T phase is dynamically unstable in free-standing conditions and it can further relax to other 1T′ phase.^61^ Due to structural distortion, spontaneous symmetry breaks down and the degeneracy of electronic states can be shifted to lower energy. 2H to 1T′ phase transition was observed that electron doping from gold, facilitated by interfacial tensile strain. They observed a tunable inverted gap (∼0.50 eV) and a fundamental gap (∼0.10 eV) in quasimetallic MoS_2_ monolayer.^62^ Owing to this inverted gap is yet observed experimentally, the source of the inverted gap is not clear.

The fundamental gap (*E*_g_) values of the pristine single layer 1T′ phase MoS_2_, MoSe_2_, WS_2_ and MoSe_2_ are observed to be ∼0.05 eV, 0.04 eV, 0.06 eV and 0.05 eV, respectively. Because the spin–orbit coupling effect could open a fundamental gap, our calculations are performed at the PBE and PBE + SOC levels. We have intriguingly observed a larger mid-infrared inverted gap (2*δ*) of the pristine single layer 1T′ phase MoS_2_ (∼0.61 eV), MoSe_2_ (∼0.77 eV), WS_2_ (∼0.24 eV) and MoSe_2_ (∼0.78 eV) at Γ-point in the 2D Brillioun zone of the distorted octahedral phase in Fig. 2. DFT calculations predicted that inverted gap and its electronic structure with low band gap might stream from the distorted octahedral phases. GW-based DFT calculations can predict a larger inverted gap due to take account the many-body effects of electron–electron interactions. It is also important to measure and understand the origin of the inverted band gap for future 2D-TMDs based device fabrication.

Raman spectroscopy has been extensively used as a tool to characterize the structure and thickness of 2D films and obtain information on their structures. Raman spectra of different phases of TMDs can therefore be used as a fingerprint of the interaction strength and other interface effects, providing a valuable insight into the interface physics of these systems. Raman shifts are directly influenced by the symmetry of the crystal lattice. Different symmetries lead to distinct vibrational modes, which are reflected as specific peaks in the Raman spectrum. Changes in crystal symmetry, such as through phase transitions, can lead to shifts or the appearance/disappearance of certain peaks. Therefore, we discuss 2H, 1T and 1T′ phases, separately. Firstly, according to the irreducible representation of 2H-phase (*D*_3h_ symmetry), the optical Raman active modes of the monolayer correspond to E^1^_2_, and A_1g_ symmetries. MoS_2_, MoSe_2_, WS_2_ and MoSe_2_ shows E^1^_2_, and A_1g_ modes at ∼376 cm^−1^ and 401 cm^−1^; ∼236 cm^−1^ and 278 cm^−1^; ∼347 cm^−1^ and 412 cm^−1^; ∼238 cm^−1^ and 244 cm^−1^, respectively (Fig. S5, ESI†).

### 2.2. Chalcogen defect dependent single layer 2H, 1T and 1T′ phase XY_2_ (X = Mo, W; Y = S, Se)

Transition metal dichalcogenides (TMDs) play a critical role in shaping the material's properties and potential quantum emitter applications. These defects are primarily associated with missing (vacancies), substituted, or displaced chalcogen atoms. The most common defects are vacancies that show that the sulfur (S) or selenium (Se) atom is missing from the lattice. These defects can occur naturally during material growth or can be intentionally introduced using techniques such as annealing, ion irradiation, or chemical treatments.

**Fig. 3:**
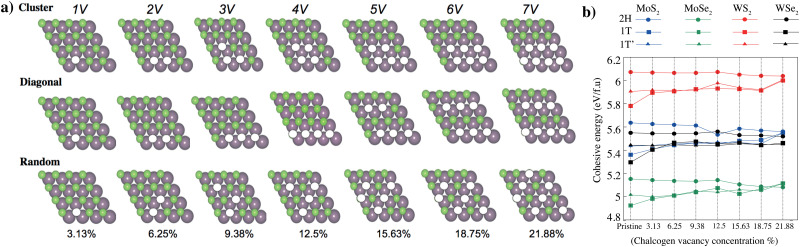
(a) DFT-optimized theoretical models of monolayer 2H-Phase TMD with different levels of chalcogen vacancy concentration. The modeled unit cell consists of 16 transition metal atoms (Mo, W) and 32 chalcognes (S, Se) atoms for the pristine stoichiometric TMD crystal. Transition metals (Mo, W) and chalcogens (S, Se) are represented with purple and green spheres, respectively. Under the assumption that chalcogen vacancies form only at the exposed surface, the percentage of chalcogen vacancy can be defined as the number of vacancies divided by the total chalcogen atoms. Multiple different configurations were considered and classified as “cluster”, “diagonal”, and “random”; based on the configuration of the chalcogen vacancies. (b) Formation energy of the 2H, 1T and 1T′ phase MoS_2_, MoSe_2_, WS_2_, and WSe_2_ monolayers as a function of the concentration of chalcogen vacancy.

**Fig. 4:**
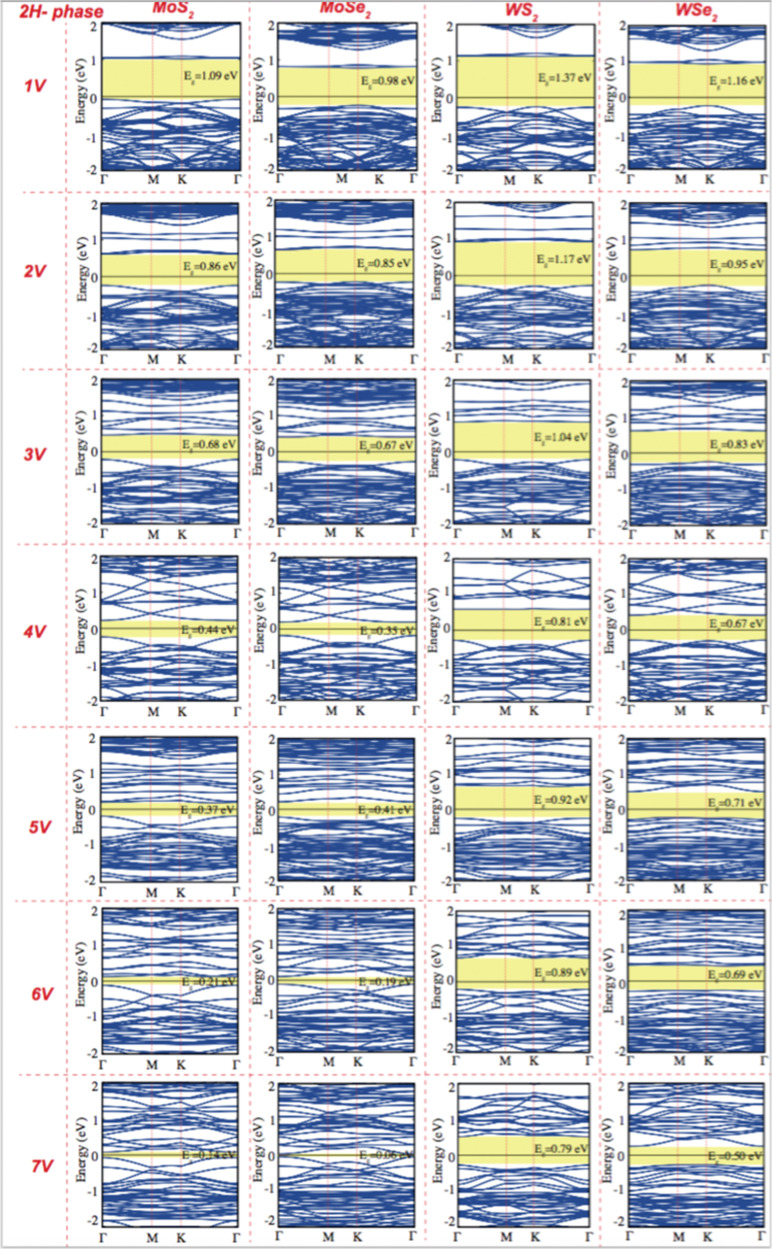
Electronic band structure of pristine single layer 2H phase MoS_2_, MoSe_2_, WS_2_, and WSe_2_ as a function of the concentration of chalcogen vacancy. The black line indicates the Fermi level at zero eV.

We have modeled a unit cell consisting of 16 transition metal atoms (Mo, W) and 32 chalcogenes (S, Se) atoms for the pristine stoichiometric TMD crystal. Under the assumption that chalcogen vacancies form only at the exposed surface, the percentage of chalcogen vacancies can be defined as the number of vacancies divided by the total chalcogen atoms. Multiple different configurations were considered and classified as “cluster”, “diagonal”, and “random”; based on the configuration of the chalcogen vacancies shown in Fig. 3(a). We have selected minimum energy cases from these three different configurations (Table 2). Chalcogen defects in TMDs have a direct impact on cohesive/formation energies, which in turn govern defect stability and concentration, and their influence on the material's overall properties. Understanding and controlling these cohesive/formation energies are crucial for optimizing TMDs for specific applications such as electronics, catalysis, or sensors. The cohesive energy of the 2H, 1T and 1T′ phase MoS_2_, MoSe_2_, WS_2_, and WSe_2_ monolayers as a function of the concentration of chalcogen vacancy are shown in Fig. 3(b) (Table S2, ESI†). While defects with low formation energies are more likely to form spontaneously during growth or under specific environmental conditions, defects with high formation energies are less likely to occur naturally and might require external energy input (*e.g.*, *via* annealing or irradiation) to form. As the concentration of chalcogen vacancies increases, the cohesive energy of 2H phase MoS_2_, MoSe_2_, WS_2_, and WSe_2_ monolayers decreases (Fig. 4), unlike the 1T and 1T′ phases. We have observed two main concepts: the first one is that the 2H phase is the most stable in all different types of TMDs. Among all TMDs, the measure of stability decreases like WS_2_, MoS_2_, WSe_2_, and MoSe_2_ monolayers. The electronic band diagrams that vary depending on the vacancy concentrations of 2H, 1T and 1T′ phase MoS_2_, MoSe_2_, WS_2_, and WSe_2_ monolayers are provided in the ESI,† Fig. S2–S4. We also further examined the thermal stability of 2H phase MoS_2_ monolayers as a function of the concentration of chalcogen vacancy by *ab initio* molecular dynamics (AIMD) simulations. Our simulation results demonstrate that these materials remain thermally stable, apart from minor distortions, the crystallinity of both phases is preserved, even at high concentrations. These materials simulation processes have been recorded and presented in the ESI,† Videos S1–S7.

The calculated total energy (*E*_tot_) values in eV for 2H, 1T and 1T phase MoS_2_, MoSe_2_, WS_2_, and WSe_2_ monolayers as a function of the concentration of chalcogen vacancy

**Table:** 

System	2H-MoS_2_			1T-MoS_2_			1T′-MoS_2_		
	Cluster	Diagonal	Random	Cluster	Diagonal	Random	Cluster	Diagonal	Random
1V	**−351.27**	**−351.27**	**−351.27**	**−341.07**	**−341.07**	**−341.07**	**−342.58**	**−342.58**	**−342.58**
2V	**−344.37**	**−344.37**	**−344.37**	−335.52	−335.52	**−336.33**	−335.63	−335.63	**−336.95**
3V	−337.50	**−337.59**	−337.35	−329.34	−329.66	**−330.83**	−329.43	−329.36	**−330.87**
4V	−330.07	**−331.22**	−330.45	−323.02	−322.91	**−324.18**	−342.48	−323.25	**−324.78**
5V	−323.26	−323.38	**−323.38**	316.35	−318.41	**−318.88**	−318.09	−317.64	**−318.18**
6V	−315.85	−316.19	**−316.19**	**−312.77**	−311.44	−311.54	−309.90	−311.05	**−311.43**
7V	−308.92	−309.37	**−309.38**	−303.50	−308.94	**−308.93**	−303.49	**−308.92**	−306.07

**Table:** 

System	2H-MoSe_2_			1T-MoSe_2_			1T′-MoSe_2_		
	Cluster	Diagonal	Random	Cluster	Diagonal	Random	Cluster	Diagonal	Random
1V	**−324.77**	**−324.77**	**−324.77**	**−317.21**	**−317.21**	**−317.21**	**−318.03**	**−318.03**	**−318.03**
2V	**−318.54**	**−318.54**	**−318.54**	**−312.72**	−312.73	−312.25	−311.88	−311.34	**−312.80**
3V	−312.36	**−312.47**	−312.12	−305.52	**−308.24**	−306.61	−306.16	−305.58	**−308.61**
4V	−305.49	**−306.99**	−305.90	−300.13	**−303.95**	−301.99	−301.16	−299.69	**−302.41**
5V	**−299.44**	−299.42	−299.31	−293.76	−295.24	**−295.96**	−294.80	−294.96	**−297.49**
6V	−292.54	**−292.77**	−292.72	−289.45	−293.22	**−291.98**	−289.51	−289.01	**−291.24**
7V	−286.33	−286.65	**−286.65**	−283.17	**−288.03**	−284.52	−284.91	**−288.01**	−284.71

**Table:** 

System	2H-WS_2_			1T-WS_2_			1T′-WS_2_		
	Cluster	Diagonal	Random	Cluster	Diagonal	Random	Cluster	Diagonal	Random
1V	**−386.01**	**−386.01**	**−386.01**	**−377.85**	**−377.85**	**−377.85**	**−378.92**	**−378.92**	**−378.92**
2V	**−378.89**	**−378.89**	**−378.89**	−370.61	**−371.62**	−371.46	−372.00	**−372.02**	−371.99
3V	−371.83	**−371.91**	−371.71	−363.45	−364.53	**−365.63**	**−365.21**	**−365.16**	−365.04
4V	−364.14	**−365.29**	−364.63	−357.93	−358.11	**−359.02**	−357.39	**−361.06**	−358.62
5V	−357.18	−357.32	**−357.33**	−350.16	**−351.99**	−351.63	−351.81	**−352.50**	−351.85
6V	−349.52	**−349.96**	−349.96	−343.76	**−344.65**	−344.62	−343.87	−344.75	**−344.91**
7V	−342.35	**−342.92**	−342.93	−337.37	**−341.34**	−341.19	−338.20	**−341.60**	−338.18

**Table:** 

System	2H-WSe_2_			1T-WSe_2_			1T′-WSe_2_		
	Cluster	Diagonal	Random	Cluster	Diagonal	Random	Cluster	Diagonal	Random
1V	**−357.62**	**−357.62**	**−357.62**	**−351.03**	**−351.03**	**−351.03**	**−338.54**	**−338.54**	**−338.54**
2V	**−351.20**	−351.20	−351.20	−345.61	**−347.62**	−346.12	−346.01	−346.01	**−346.74**
3V	−344.88	**−347.98**	−344.65	−338.55	**−341.78**	−341.01	−340.06	−339.61	**−340.28**
4V	−337.74	**−339.31**	−338.30	−334.08	−333.41	**−334.59**	−333.49	−333.47	**−334.01**
5V	−331.57	**−331.65**	−331.65	−326.62	−328.37	**−328.85**	−326.79	−326.79	**−328.46**
6V	−324.49	−324.87	**−325.18**	−322.98	**−321.69**	**−321.69**	−320.30	−320.11	**−321.96**
7V	−318.23	**−318.56**	−318.56	−314.12	**−316.25**	−316.24	−313.70	−314.12	**−315.76**

Creating vacancies in TMDs leads to the formation of defect bands that lead to a significant reduction in band gap energy. While pristine monolayer 2H TMDs exhibit a band gap of approximately 1.43 to 1.81 eV, creating vacancies at varying concentrations reduces the band gap to values ranging from 0.14 to 1.37 eV, as shown in Fig. 4. The emergence of mid-gap states caused by vacancies is a key factor in this reduction, making it especially important for quantum emitter applications. The energy range of the visible region typically changed from 1.65 eV to 3.1 eV. Pristine 2H-phase TMDs are observed to shift from the visible region to the IR region, and this tunability highlights the potential of defect dependent TMDs for applications in quantum technologies and photonic devices.

## 3. Conclusions

In summary, we investigate the structural and electronic properties of pristine 2H, 1T and 1T phases of TMDs using DFT. Our results reveal the distinct electronic properties across their various phases (2H, 1T, and 1T) due to their different coordination structures and stacking orders. The introduction of chalcogen vacancies significantly reduces the band gap in 2H, 1T, and 1T′ phases of MoS_2_, WS_2_, MoSe_2_, and WSe_2_, and can lead to transitions from direct to indirect band gaps, as well as the emergence of semi-metallic or magnetic band structures. Interestingly, while the 2H phase is well-studied for its catalytic and energy-related applications, the 1T phase, despite its higher formation energy, offers enhanced charge transfer efficiency and is metastable, allowing it to transition into the 2H phase under certain conditions. However, a comprehensive understanding of these phases, especially in the presence of chalcogen vacancies, is necessary for practical applications. The chalcogen defects, particularly vacancies, significantly influence the properties and potential applications of TMDs. These defects affect the cohesive and formation energies, which determine the stability, concentration, and overall impact on the material's behavior. Our study indicates that the stability of the 2H phase decreases as the concentration of chalcogen vacancies increases. Among the TMDs analyzed (MoS_2_, WS_2_, MoSe_2_, and WSe_2_), the 2H phase is consistently the most stable, with stability following the trend WS_2_ > MoS_2_ > WSe_2_ > MoSe_2_. Our findings provide important insights for the experimental exploration and engineering of TMD defects, supporting the development of advanced spintronic, electronic, optoelectronic and quantum emitting devices.

## Author contributions

B. A. H.: conceptualization, methodology, software, validation, formal analysis, investigation, resources, data curation, writing – original draft, writing – review and editing, visualization, and supervision. M. C. O.: conceptualization, methodology, resources, writing – original draft, writing – review and editing, visualization, supervision, project administration, and funding acquisition.

## Data availability

Our article does not require legal or ethical confidentiality requirements. Data for this article, including Fig. 1 are available at VESTA at https://jp-minerals.org/vesta/en/download.html. Data for this article, including Fig. 2–4 are available at VASP at https://www.vasp.at/. The data supporting this article have been included as part of the ESI.†

## Conflicts of interest

There are no conflicts to declare.

## Supplementary Material

CP-027-D4CP04017A-s001

CP-027-D4CP04017A-s002

CP-027-D4CP04017A-s003

CP-027-D4CP04017A-s004

CP-027-D4CP04017A-s005

CP-027-D4CP04017A-s006

CP-027-D4CP04017A-s007

CP-027-D4CP04017A-s008

CP-027-D4CP04017A-s009

CP-027-D4CP04017A-s010

CP-027-D4CP04017A-s011

CP-027-D4CP04017A-s012

CP-027-D4CP04017A-s013

CP-027-D4CP04017A-s014
