# Supplementary material for: Defect dependent electronic properties of two-dimensional transition metal dichalcogenides (2H, 1T, and 1T′ phases)
Source: Phys Chem Chem Phys. 2024 Dec 6;27(4):1809–18. doi: 10.1039/d4cp04017a (PMC11698123; doi:10.1039/d4cp04017a)
Supplement: CP-027-D4CP04017A-s006 [file CP-027-D4CP04017A-s006.pdf]

Supplementary Information (SI) for Physical Chemistry Chemical Physics.
This journal is © the Owner Societies 2024

Journal Name

ARTICLE TYPE

Cite this: DOI: 00.0000/xxxxxxxxxx

Supplementary Information: Defect dependent electronic properties of two-dimensional transition metal dichalcogenides (2H, 1T, 1T' phase)

Berna Akgenc Hanedar [a,b] and Mehmet Cengiz Onbaşlı [b,c]

Received Date
Accepted Date

DOI: 00.0000/xxxxxxxxxx

1 Pristine monolayer 2H, 1T and 1T' TMD phases

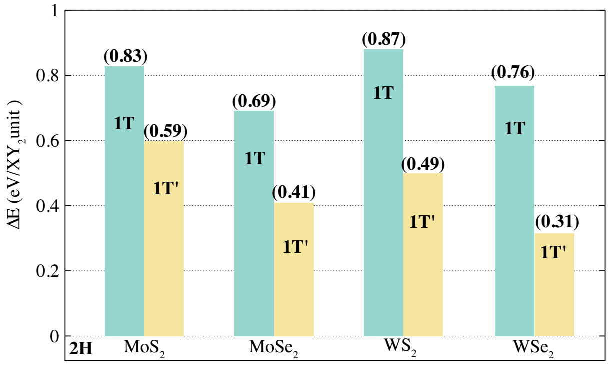

Fig. 1 The ground-state energy difference (per XY_2 (X = Mo, W; Y = S, Se) unit) between the 2H, 1T and 1T' phases (the energy of the 2H monolayer is set as the reference, indicated by the dashed line at zero).

2 Chalcogen defect dependent single layer 2H, 1T and 1T' phase XY_2 (X = Mo, W; Y = S, Se)

Table 1 The calculated cohesive (E_{coh}) and formation (E_f) energy values in eV/atom for the TMD layers.

2H-MoS_2	1V	2V	3V	4V	5V	6V	7V
E_{coh}	5.626	5.618	5.612	5.533	5.585	5.569	5.560
E_f	-0.748	-0.706	-0.664	-0.630	-0.561	-0.503	-0.452
1T-MoS_2	1V	2V	3V	4V	5V	6V	7V
E_{coh}	5.409	5.443	5.462	5.454	5.480	5.487	5.550
E_f	-0.531	-0.531	-0.514	-0.469	-0.456	-0.421	-0.441
1T'-MoS_2	1V	2V	3V	4V	5V	6V	7V
E_{coh}	5.441	5.457	5.463	5.469	5.464	5.455	5.549
E_f	-0.563	-0.544	-0.515	-0.469	-0.440	-0.389	-0.441
2H-$MoSe_2$	1V	2V	3V	4V	5V	6V	7V
E_{coh}	5.142	5.136	5.133	5.143	5.105	5.087	5.081
E_f	-0.552	-0.507	-0.465	-0.434	-0.353	-0.290	-0.237
1T-$MoSe_2$	1V	2V	3V	4V	5V	6V	7V
E_{coh}	4.982	5.009	5.039	5.073	5.024	5.068	5.114
E_f	-0.391	-0.381	-0.371	-0.365	-0.273	-0.271	-0.271
1T'-$MoSe_2$	1V	2V	3V	4V	5V	6V	7V
E_{coh}	4.999	5.011	5.047	5.038	5.060	5.050	5.114
E_f	-0.408	-0.383	-0.379	-0.330	-0.308	-0.254	-0.270
2H-WS_2	1V	2V	3V	4V	5V	6V	7V
E_{coh}	6.068	6.065	6.065	6.073	6.050	6.040	6.038
E_f	-0.468	-0.415	-0.363	-0.317	-0.237	-0.167	-0.102
1T-WS_2	1V	2V	3V	4V	5V	6V	7V
E_{coh}	5.894	5.907	5.925	5.930	5.926	5.914	5.999
E_f	-0.295	-0.257	-0.224	-0.174	-0.113	-0.041	-0.064
1T'-WS_2	1V	2V	3V	4V	5V	6V	7V
E_{coh}	5.441	5.457	5.463	5.469	5.464	5.455	5.549
E_f	-0.563	-0.544	-0.515	-0.469	-0.440	-0.389	-0.441
2H-WSe_2	1V	2V	3V	4V	5V	6V	7V
E_{coh}	5.544	5.542	5.545	5.560	5.529	5.526	5.518
E_f	-0.232	-0.177	-0.124	-0.080	0.010	0.077	0.152
1T-WSe_2	1V	2V	3V	4V	5V	6V	7V
E_{coh}	5.404	5.464	5.474	5.453	5.464	5.443	5.462
E_f	-0.092	-0.099	-0.053	0.026	0.075	0.160	0.208
1T'-WSe_2	1V	2V	3V	4V	5V	6V	7V
E_{coh}	5.138	5.445	5.440	5.440	5.455	5.449	5.450
E_f	0.173	-0.080	-0.019	0.039	0.084	0.154	0.221

[a] *Department of Physics, Kirklareli University, Kirklareli, 39100, Türkiye Fax: +90 288 246 17 33; Tel: +90 288 246 17 34; E-mail: berna.akgenc@klu.edu.tr*
[b] *Department of Physics, Koc University, Rumelifeneri Yolu, Sariyer 34450 Istanbul, Turkey E-mail: bhanedar@ku.edu.tr*
[c] *Department of Electrical & Electronics Engineering, Koc University, Rumelifeneri Yolu, Sariyer 34450 Istanbul, Turkey Tel: +90 212 338 1711; E-mail: monbasli@ku.edu.tr*

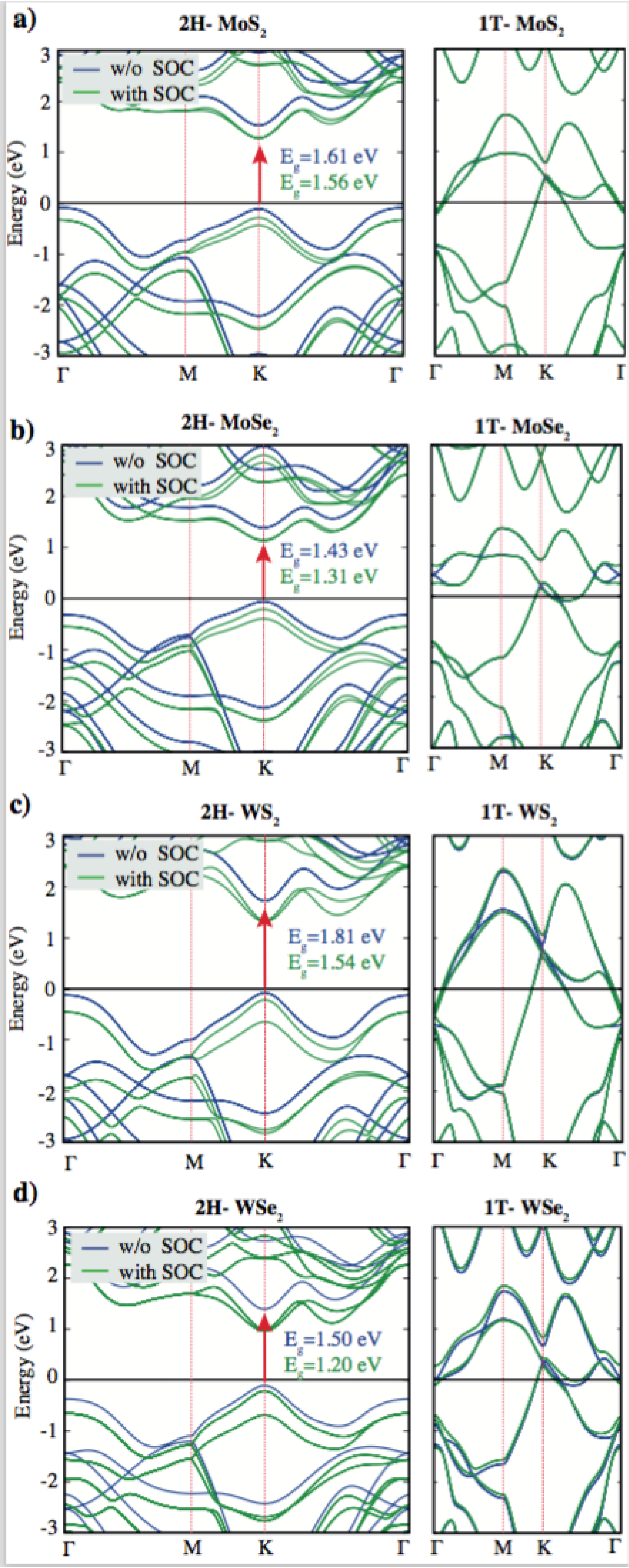

Fig. 2 Band structure of pristine single layer 2H and 1T phase XY_2 (X = Mo, W; Y = S, Se), PBE band structure is shown with a blue line and the PBE+SOC band structure is presented with a green line. The black line indicates the Fermi level at zero eV.

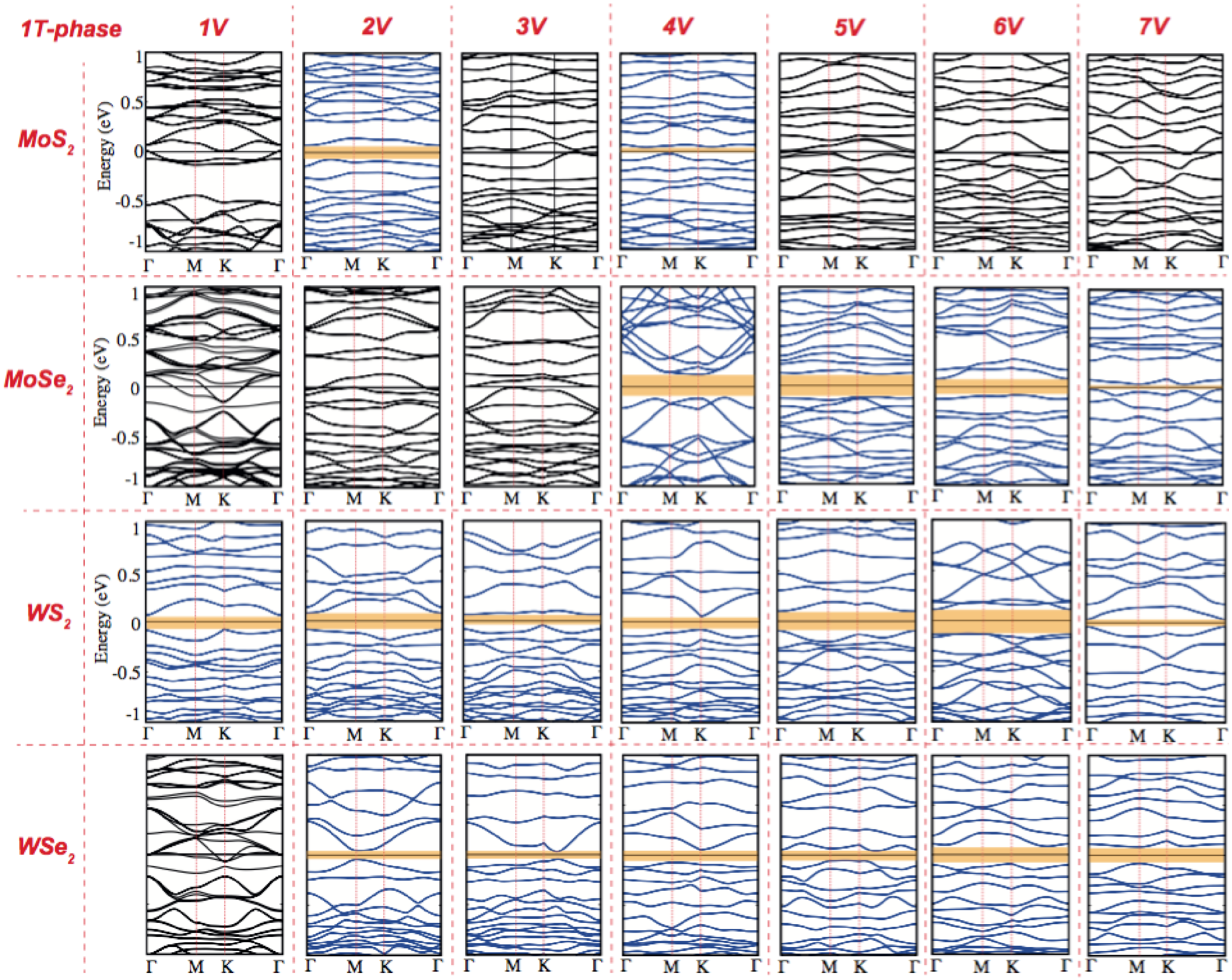

Fig. 3 Electronic band structure of pristine single layer 1T phase MoYM_2 (Y = S,Se)

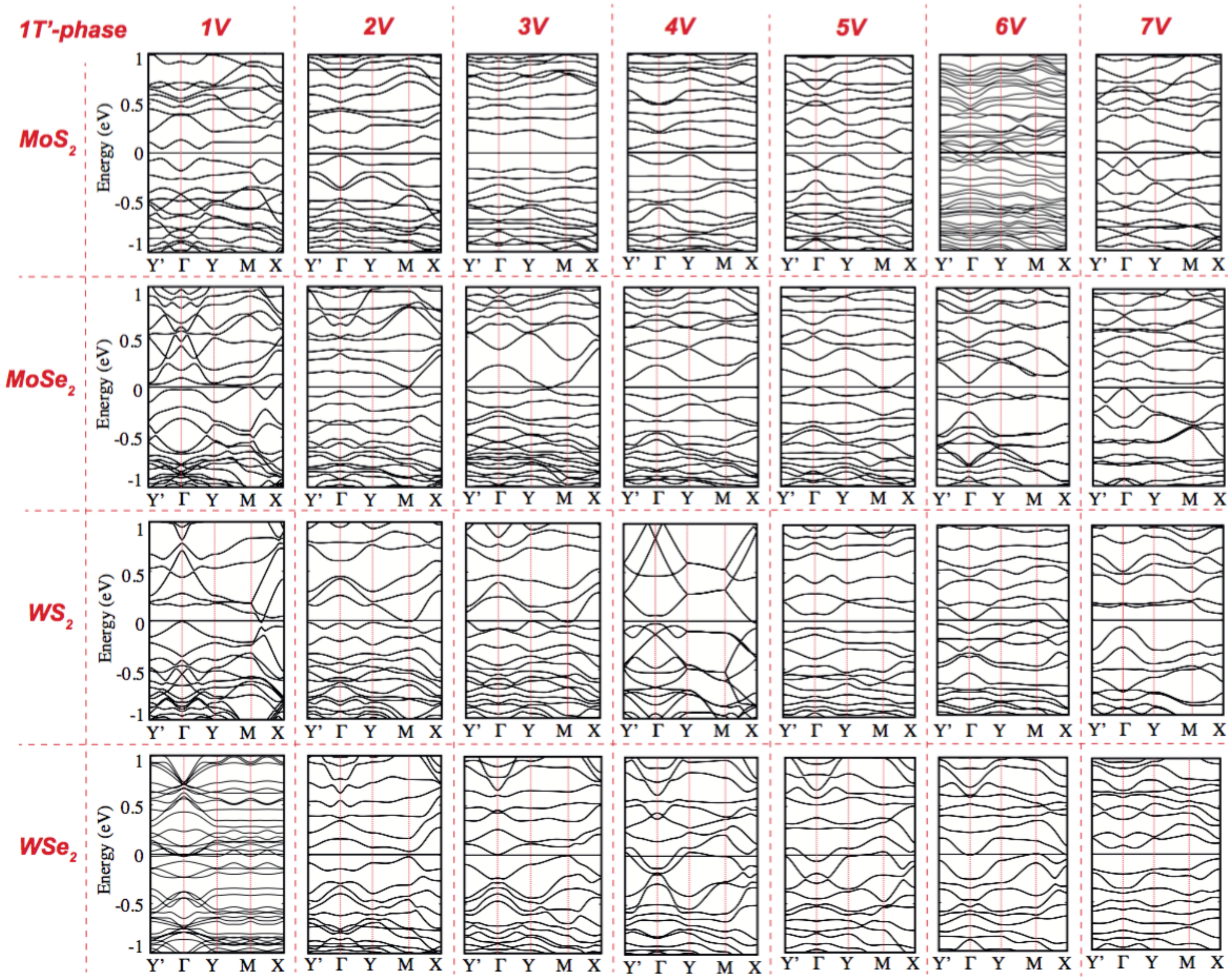

Fig. 4 Electronic band structure of pristine single layer 1T' phase MoY_2 (Y = S,Se)

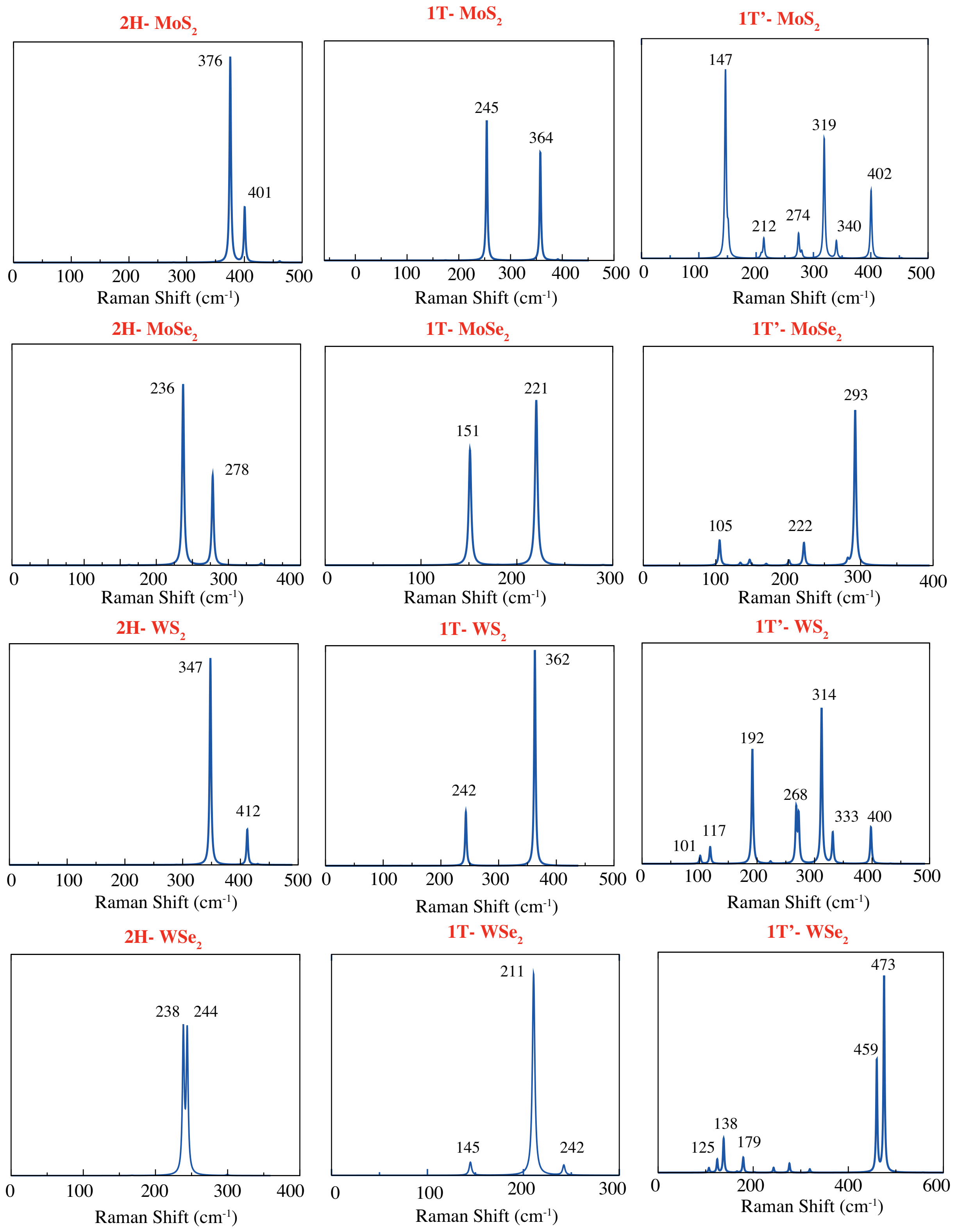

Fig. 5 Raman spectrum of pristine single layer 2H, 1T and 1T' phase XY_2 (X = Mo,W Y = S,Se)
